# Sepsis induces albuminuria and alterations in the glomerular filtration barrier: a morphofunctional study in the rat

**DOI:** 10.1186/cc10559

**Published:** 2011-11-22

**Authors:** Chiara Adembri, Eleonora Sgambati, Luca Vitali, Valentina Selmi, Martina Margheri, Alessia Tani, Laura Bonaccini, Daniele Nosi, Anna L Caldini, Lucia Formigli, Angelo R De Gaudio

**Affiliations:** 1 Department of Medical and Surgical Critical Care, Section of Anesthesiology and Intensive Care, University of Florence, Azienda Ospedaliero-Universitaria Careggi, Largo Brambilla, 3, 50134 Florence, Italy; 2 Department of Sciences and Environmental Technologies, University of Molise, Contrada Fonte Lappone, 86090 Pesche (Isernia), Italy; 3 Department of Anatomy, Histology, Forensic Medicine and Physiological Sciences, University of Florence, Largo Brambilla, 3, 50134 Florence, Italy; 4 Clinical Chemistry Laboratories, Azienda Ospedaliero-Universitaria Careggi, Largo Brambilla, 3 50134 Florence, Italy

## Abstract

**Introduction:**

Increased vascular permeability represents one of the hallmarks of sepsis. In the kidney, vascular permeability is strictly regulated by the 'glomerular filtration barrier' (GFB), which is comprised of glomerular endothelium, podocytes, their interposed basement membranes and the associated glycocalyx. Although it is likely that the GFB and its glycocalyx are altered during sepsis, no study has specifically addressed this issue. The aim of this study was to evaluate whether albuminuria -- the hallmark of GFB perm-selectivity -- occurs in the initial stage of sepsis and whether it is associated with morphological and biochemical changes of the GFB.

**Methods:**

Cecal ligation and puncture (CLP) was used to induce sepsis in the rat. Tumor necrosis factor (TNF)-alpha levels in plasma and growth of microorganisms in the peritoneal fluid were evaluated at 0, 3 and 7 hours after CLP or sham-operation. At the same times, kidney specimens were collected and structural and ultrastructural alterations in the GFB were assessed. In addition, several components of GFB-associated glycocalyx, syndecan-1, hyluronan (HA) and sialic acids were evaluated by immunofluorescence, immunohistochemistry and lectin histochemistry techniques. Serum creatinine and creatinine clearance were measured to assess kidney function and albuminuria for changes in GFB permeability. Analysis of variance followed by Tukey's multiple comparison test was used.

**Results:**

Septic rats showed increased TNF-alpha levels and growth of microorganisms in the peritoneal fluid. Only a few renal corpuscles had major ultrastructural and structural alterations and no change in serum creatinine or creatinine clearance was observed. Contrarily, urinary albumin significantly increased after CLP and was associated with diffuse alteration in the glycocalyx of the GFB, which consisted in a decrease in syndecan-1 expression and in HA and sialic acids contents. Sialic acids were also changed in their structure, exhibiting a higher degree of acetylation.

**Conclusions:**

In its initial phase, sepsis is associated with a significant alteration in the composition of the GFB-associated glycocalyx, with loss of GFB perm-selectivity as documented by albumin leakage into urine.

## Introduction

The increased vascular permeability due to the inflammatory response that occurs during sepsis causes some of the most frequent clinical features of sepsis itself, such as hypoalbuminemia, edema, hypovolemia and altered drug distribution [1-5]. In the kidney glomeruli, vascular permeability is strictly regulated by a complex structure named the 'glomerular filtration barrier' (GFB), which is comprised of glomerular endothelium, podocytes and their interposed basement membranes: its integrity prevents the passage of albumin and high weight endogenous molecules in the urine [6,7]. One contribution to the perm-selective properties of the GFB is provided by the glycocalyx, a network of glycoproteins, proteoglycans and soluble components [8] which lines the extracellular surface of all cells, including the luminal surface of endothelial cells and the surface of podocytes of the GFB [9-11]. Sialic acids, heparan sulfate proteoglycans and hyaluronan (HA) are among the most important glycocalyx components [12-15]. Sialic acids are a large family of nine carboxylated sugars that, because of their size, negative charge and frequent terminal location in glycoconjugate oligosaccharidic chains, are responsible for membrane stability and modulation of several intercellular and/or intermolecular phenomena [16-21]. This role is due not only to their presence or absence but also to their chemical structure, because addition of one or more O-acetyl esters to hydroxyl groups and/or change in the type of link to the underlying sugar chains greatly modifies their functional properties [17,22-25]. Sialic acids of the GFB have recently been demonstrated to play an important role in maintaining its structure and in regulating its filtration properties [10,26-30]. Also syndecan-1, an integral heparan sulfate proteoglycan component that has one to three glycosaminoglycan (GAG) molecules attached to its core [12], seems to participate in the maintenance of the structural integrity of the GFB glycocalyx and of its functional properties [9]. Because a loss of HA has been associated with pathological conditions characterized by an increased vascular permeability such as diabetes [31,32] and ischemia-reperfusion [33], the HA content of the GFB might be decreased during sepsis as well.

Although kidney injury occurs very frequently during sepsis, its pathophysiology is not that well understood [34,35]. Most studies have focused on alterations of perfusion whereas the role of changes in GFB structure and/or function have scarcely been investigated, even though they are likely to occur as suggested by the early appearance of albuminuria in postoperative patients who evolve to sepsis compared to those having a regular postoperative course [36]. Acute endotoxemia models are also associated with changes in GFB properties and glycocalyx dysfunction [37-41]. However, to our knowledge, no study has specifically addressed this issue in experimental models reliably mimicking human sepsis [42].

The aim of this study was therefore to evaluate whether albuminuria - the hallmark of GFB dysfunction - occurs in the early stage of a clinically relevant, controlled rat model of polymicrobial sepsis (the Cecal Ligation and Puncture (CLP) model) and whether it is associated with changes in structural, ultrastructural and biochemical composition of the GFB.

## Materials and methods

### Animals and experimental protocol

Experiments were performed on adult male Sprague-Dawley rats (n = 34; Harlan, Udine, Italy) weighing 300 to 350 g, housed three per cage and maintained in a controlled environment (temperature 22 + 1°C and 12-hour light:12-hour dark cycle) with unlimited access to food and water. The experimental protocol was approved by the Commission for Animal Experimentation of the Ministry of Health, Rome, Italy, according to Italian and European Guidelines for Animal Care and Experimentation, DL 116/92, application of the European Communities Council Directive (86/609/EEC).

After acclimatization, animals were assigned to one of the following experimental groups: sham-operated (n = 15) as controls, and Cecal Ligation and Puncture (CLP; n = 19) as the experimental sepsis group. All rats were anesthetized with sodium pentobarbital (65 mg/kg, i.p.), positioned on a homeothermic heating pad, and body temperature was maintained between 36.5°C and 37.5°C. After anesthesia the rats underwent transurethral catheterization with a PE50 tubing passing into the bladder in order to empty it. The catheter was secured with a hitch suture onto the lower abdomen, oriented in a manner which did not alter the normal urethral axis. CLP or sham surgery was then performed. After surgery, rats were returned to their home cages in the same environmental conditions. Observations were made at times 0 (baseline), 3 and 7 hours after surgical procedures.

### The CLP-induced sepsis

CLP was used to induce sepsis [42]. Briefly, a 3-cm midline laparotomy was made first through the skin and then through the *linea alba *to expose the cecum with the adjoining intestine. The cecum was exteriorized and ligated at its base, below the ileocecal valve in a non-obstructing manner with a 3.0 silk. Then the ligated cecum was punctured with a 16-gauge needle, allowing entrapped fecal material to leak into the normally sterile peritoneal cavity. The cecum was then repositioned in the peritoneal cavity and the abdomen was closed in two layers. Sham-operated animals received laparotomy only. Samples of peritoneal fluid were taken at two experimental times (3 and 7 hour) and cultured for the growth of Gram-positive/Gram-negative isolates. Samples were incubated on Mannitol Salt Agar for 24 hours at 37°C for Gram-positive strains and layered on MacConkey Agar III for 24 hours at 37°C for Gram-negative culture. Species identification was determined by the automated Vitek2 system (bioMérieux, Marcy l'Etoile, France), using a panel Gram-Positive (GP) card.

To demonstrate the occurrence of the inflammatory response, TNF-α levels in systemic blood were measured. Blood samples were drawn via cardiac puncture at the same times (0, 3, and 7 hours) in the two experimental groups (at least n = 5 animals per group each time). The blood was immediately centrifuged at 4,000 rpm for 15 minutes at 4°C, plasma was collected, divided into aliquots and stored at -80°C until assayed. The TNF-α plasma level was measured using an enzyme-linked immunosorbent assay according to the manufacturer's instructions (Bender MedSystems, Vienna, Austria). The sensitivity of the assay was 11 pg/mL.

### Assessment of renal and GFB damage during sepsis

#### Functional assessment of damage

Creatinine serum levels and creatinine clearance (calculated as urinary creatinine/seric creatinine*urinary volume/minute and expressed as mL/min/100 g body weight) were assessed as an index of acute renal injury. Albumin urinary content was measured as an index of GFB change in perm-selectivity and expressed as albumin/urinary creatinine ratio to normalize for urinary volume. Urine and blood were sampled at 0, 3, 7 hours following CLP or sham-operation (at least n = 4 rats per group each time). Serum and urinary creatinine levels were measured by an enzymatic method on an ADVIA 2400 Chemistry System Analyzer (Siemens Healthcare, Milan, Italy). Total serum proteins were measured by using biuret reagent and total urinary proteins by the Pyrogallol Red colorimetric assay (ADVIA 2004, Siemens Healthcare, Milan, Italy). Urinary protein electrophoresis was performed on agarose gel followed by violet acid on Hydrasys (SEBIA, Lisses, France); each sample was run in duplicate. Albumin concentrations were derived after scan densitometry of the gel by proportion with urinary total proteins. In order to evaluate the different origin of urinary proteins (glomerular or tubular), urine samples were analyzed by agarose/sodium dodecyl sulfate (SDS) gel electrophoresis painted with violet acid.

#### Morphological assessment of damage

Animals were euthanized with an overdose of pentobarbital (200 mg/kg, i.p.) at baseline and 3 and 7 hours after CLP or sham-operation and processed in different ways according to the histological technique used for morphological analysis.

##### Structural and ultrastructural analysis

Kidney samples were fixed in a solution containing 2% glutaraldehyde, 2% sucrose, 0.1 mol/L sodium cacodylate phosphate and 2% lanthanum nitrate for 4 hours at room temperature, and postfixed in 1% osmium tetroxide in 0.1 M phosphate buffer, pH 7.4, for 1 hour at 4°C for light microscopy and transmission electron microscopy. Then the specimens were dehydrated in graded acetone, passed through propylene-oxide and embedded in Epon 812. Semi-thin sections, 2 μm thick, were stained with toluidine blue-sodium tetraborate and observed under light microscopy. For quantitative analysis, the number of damaged renal corpuscles was evaluated by counting 10 random 600625 μm^2 ^optical square fields (40 × ocular) under an inverted phase-contrast Nikon DIAPHOT 300 microscope (NIKON, Melville, NY, USA) in each experiment. The number of damaged corpuscles counted by two different observers was expressed as the percentage of the total renal corpuscles. Ultrathin sections were stained with uranyl acetate and alkaline bismuth subnitrate and then examined under a transmission electron miscroscoey (Jeol 1010, Tokyo, Japan) at 80 kV.

##### Confocal immunofluorescence

After the pentobarbital overdose, a midline incision was made in the abdomen and thorax of 16 rats; the kidneys were immediately fixed by transcardial perfusion by flushing with phosphate-buffered saline (PBS) for 1 minute followed by 4% paraformaldehyde in PBS buffer for 3 minutes. All solutions were maintained at pH 7.4 at 4°C. Kidneys were removed and post-fixed in 4% paraformaldehyde in PBS overnight, then transferred to PBS containing 30% sucrose and finally frozen at -80°C. For immuno-staining, cryostat sections, 10 μm thick, were permeabilized with cold acetone for 10 minutes, blocked with a solution containing 0.5% BSA (Sigma-Aldrich, St. Louis, MO, USA) and 0.2% gelatin in PBS for 30 minutes, and incubated at 4°C overnight with primary polyclonal antibody anti-syndecan-1 (1:50, Santa Cruz Biotechnology, Santa Cruz, CA, USA) followed by incubation for 1 hour at room temperature with goat anti-rabbit Alexa Fluor 488-conjugated immunoglobulin G (IgG) (1:200, Molecular Probes, Eugene, OR, USA). For HA staining, cryostat sections were blocked using 3% fetal bovine serum (FBS) in PBS for 1 hour at room temperature, incubated with (10 μg/mL) biotinylated HA binding protein (bHABP) (kindly provided by Manuela Viola, Insubria University, Varese, Italy) and visualized with Alexa Fluor 488-conjugated streptavidin (1:100, Molecular Probes). The stained sections were then rinsed and mounted with an anti-fade mounting medium (Biomeda Gel mount, Electron Microscopy Sciences, Foster City, CA, USA). Negative controls were performed by replacing the primary antibody with non-immune mouse serum or by incubating with bHABP after digestion with hyaluronidase from Streptomyces at 37°C for 2 hours (provided by Manuela Viola). Nuclei counterstaining was performed with propidium iodide (PI, 1:30; Molecular Probes). Sections were examined with a Leica TCS SP5 confocal laser scanning microscope (Leica Microsystem, Mannheim, Germany) equipped with a HeNe/Argon laser source for fluorescence measurements. Fluorescence was collected using a Leica PlanApo × 63 oil-immersion objective. Optical sections (1024 × 1024 pixels, pixel size 200 nm × 200 nm) at intervals of 400 nm were obtained and superimposed to create a single composite. To quantify syndecan-1 expression and HA content, densitometric analysis of the intensity of the fluorescence signals was performed on digitized images using ImageJ software (National Institute of Health, NIH).

##### Lectin histochemistry

After the pentobarbital overdose, a midline incision was made in the abdomen, and kidney (n = 18 rats) specimens were fixed in Carnoy's fluid and routinely processed to obtain 6 μm-thick paraffin sections. Two methodologies were used for lectin histochemistry: the 'direct' technique and the 'indirect' one. In the 'direct' technique, *Maackia amurensis *agglutinin (MAA) and *Sambucus nigra *agglutinin (SNA) were used to identify and differentiate between sialic acids linked α-2,3 and α-2,6 to galactose or galactosamine, respectively (Neu5Ac(α-2→3)Gal, Neu5Ac(α-2→6)Gal/GalNAc) [18,43-45]. In the second methodology, the 'indirect' technique, peanut agglutinin (*Arachis hypogaea*) (PNA), combined with neuraminidase digestion, deacetylation and differential oxidation to reveal acetylic groups, was used to investigate the expression of sialic acid linked to D-Gal(β1→3)-D-GalNAc, and the structure of sialic acids [18,45].

Detailed information on lectin histochemistry can be found in the Additional file 1: 'Lectin Histochemistry'.

### Statistical analysis

All values were tested for normality distribution and were expressed as mean ± standard error of the mean (SEM). To assess differences among groups, analysis of variance (ANOVA) followed by Tukey's multiple comparison test were used. Data analysis was performed using GraphPad Prism 5.0 (GraphPad Software, La Jolla, CA, USA). *P *values <0.05 were considered statistically significant.

## Results

No rats died because of technical errors or during the experimental time period, therefore all animals were included in the analysis.

Rats subjected to CLP exhibited classic signs of sepsis, including piloerection, tachypnea, diarrhea, periorbital exudates and lethargy from the first hours after CLP. Baseline TNF-α levels were similar in sham-operated and CLP-treated rats. TNF-α plasma levels were significantly increased at 3 hours after CLP compared to sham-operated animals (17.5 ± 6.2 ng/mL versus 9 ± 2.5; P <0.05 CLP versus sham-operated, at least n = 5 rats each group) and remained significantly higher at 7 hours after CLP (20.7 ± 5.6 versus 8.7 ± 2.1; *P *<0.05 versus sham-operated, at least n = 5 rats each group).

Peritoneal inflammation and purulent ascites were observed when the abdomen was reopened for kidney specimen collection at 3 and 7 hours after CLP. Cultured peritoneal fluid revealed polymicrobial flora (>10^4 ^Colony Forming Units/mL). The most frequently isolated microorganisms were *Escherichia coli *(72%), *Enterococcus faecalis *(43%), *Streptococcus viridans *(15%), and coagulase-negative staphylococci (72%).

### Functional damage

**Figure 1:**
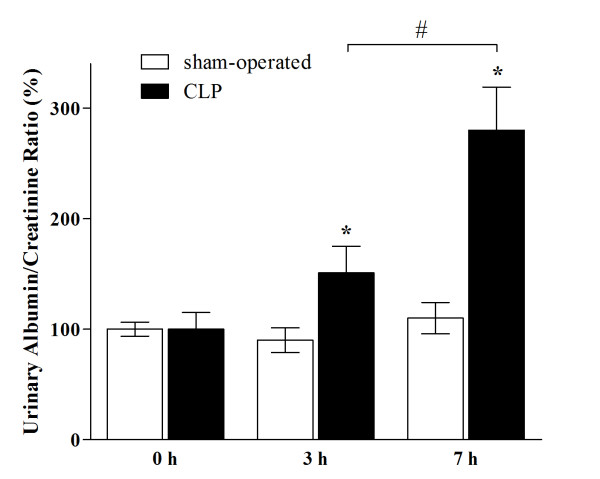
**GFB permeability to albumin was increased after sepsis**. Sepsis induced a dramatic increase in the amount of albumin measured in urine when compared to sham-injury (* *P *<0.05 CLP versus sham-operated at 3 and 7 hours, n = at least 5 rats each group). Albuminuria is expressed as urinary albumin/creatinine ratio to normalize the values for urinary volume. Data are expressed as a percentage compared to baseline. CLP, cecal ligation and puncture; n, number.

Creatinine serum levels were not different in septic animals at 3 and 7 hours after surgery as compared to sham-operated ones (0.22 ± 0.01 mg/dL in sham-operated animal versus 0.22 ± 0.03 mg/dL at 3 hours and 0.26 ± 0.04 mg/dL at 7 hours in septic rats). Similarly, creatinine clearance remained stable at 3 and 7 hours after CLP (sham-operated 0.89 ± 0.03 mL/min/100 g; CLP 0.81 ± 0.42 and 0.82 ± 0.08 mL/min/100 g at 3 and 7 hours, respectively; ns versus sham-operated). On the contrary, whereas albumin/urinary creatinine ratio remained unchanged during the study period in sham-operated animals, it increased in CLP animals by 152% and 288% at 3 h and 7 hours, respectively (for details see Figure 1). Data obtained by SDS electrophoresis clearly indicated that proteins present in the urine were limited to albumin (derived from alteration in GFB permeability) and proteins of lower molecular weight (likely 'tubular proteins', which are normally filtered by the GFB but usually reabsorbed by tubular cells).

### Morphological assessment of damage

#### Microscopy and confocal analysis

We next assessed whether albuminuria observed in the septic rats was associated with structural and ultrastructural alterations of renal corpuscles and of the GFB.

**Figure 2:**
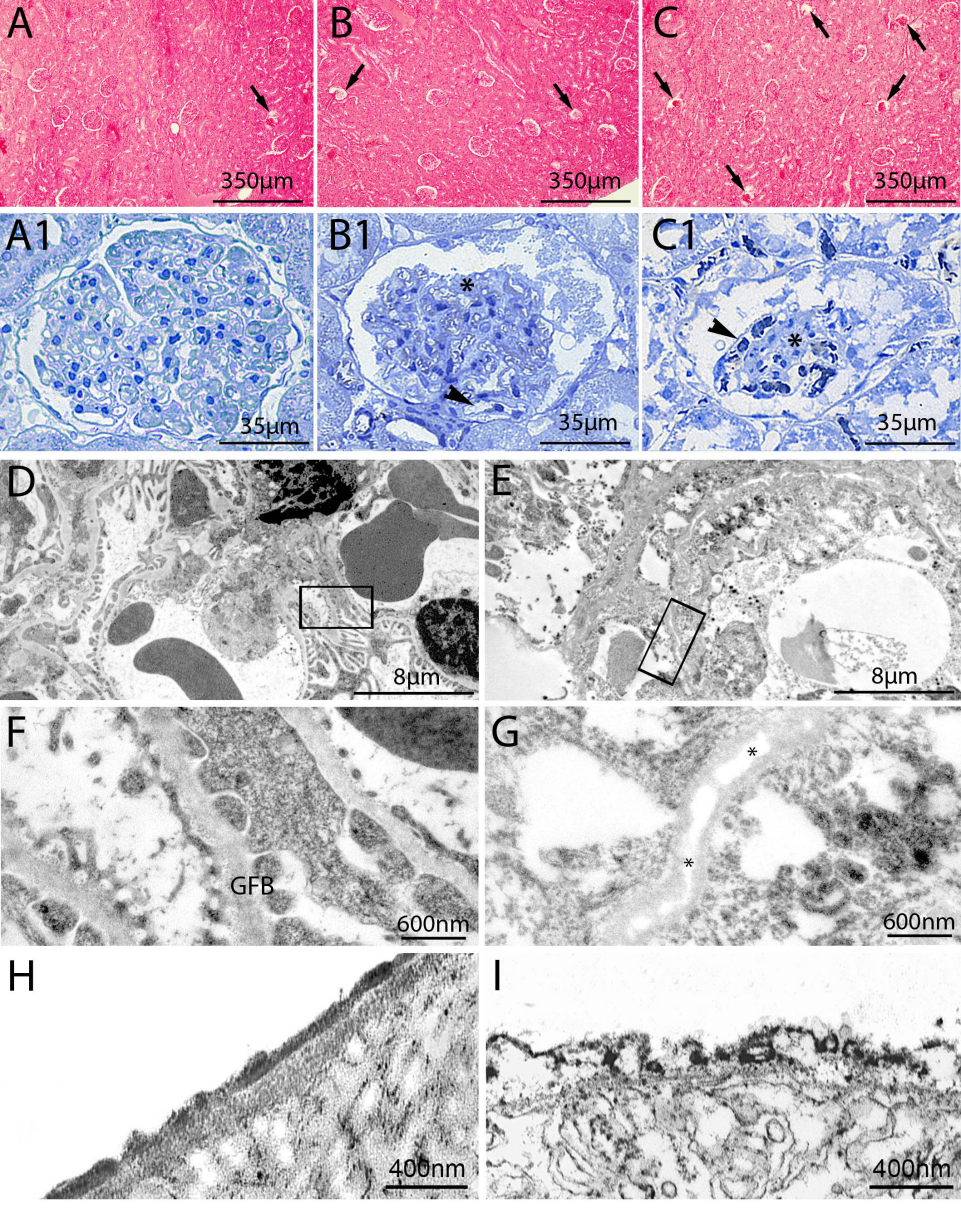
**Sepsis caused structural and ultrastructural changes in the renal corpuscles**. Representative image of light and transmission electron microscopic image of cortical renal corpuscle in sham-operated (**A, A1, D, F, H**) and septic rats (**B, C, B1, C1, E, G, I**). In septic rats some renal corpuscles had a wrinkled appearance and were much smaller (panel B, arrows); this phenomenon was more evident at 7 hours after CLP (panel C, arrows). Altered renal corpuscles of CLP rats, when compared with healthy ones (panel A1), showed enlargement of the filtration chamber and mesangial expansion with increased matrix deposition (panels B1 and C1, asterisks) and distorted capillaries (panels B1 and C1, arrowheads). At an ultrastructural level, podocytes had a normal appearance and well-developed foot processes in sham-operated rats (panel D) and the basement membrane appeared homogeneous (panel F). In contrast, sepsis caused ultrastructural modifications in the basement barrier of wrinkled corpuscles, which appeared thinner and showed focal loss of matrix components (panels E and G, asterisks). In the same corpuscles, podocytes appeared severely damaged with loss of foot processes. In sham-operated rats the endothelial glycocalyx appeared mostly intact and homogeneous (panel H), whereas in septic rats it was severely disrupted (panel I). CLP, cecal ligation and puncture.

Major changes were encountered only in a few renal corpuscles (approximately 6% to 12% of the total at 3 and 7 hours of sepsis, respectively, *P *<0.05 CLP versus sham-operated). This damage consisted mainly in wrinkled corpuscles (Figure 2, panels A, B and C), enlarged filtration chambers associated with a mild expansion of the mesangial matrix and in the presence of microthrombi in the glomerular capillaries (Figure 2, panels B1, C1).

TEM analysis of these renal corpuscles showed marked modifications of the GFB, with shedding of podocytes, loss of the endothelial cell lining (Figure 2, panel E) and massive disruption of the endothelial glycocalyx (Figure 2, panel I).

**Figure 3:**
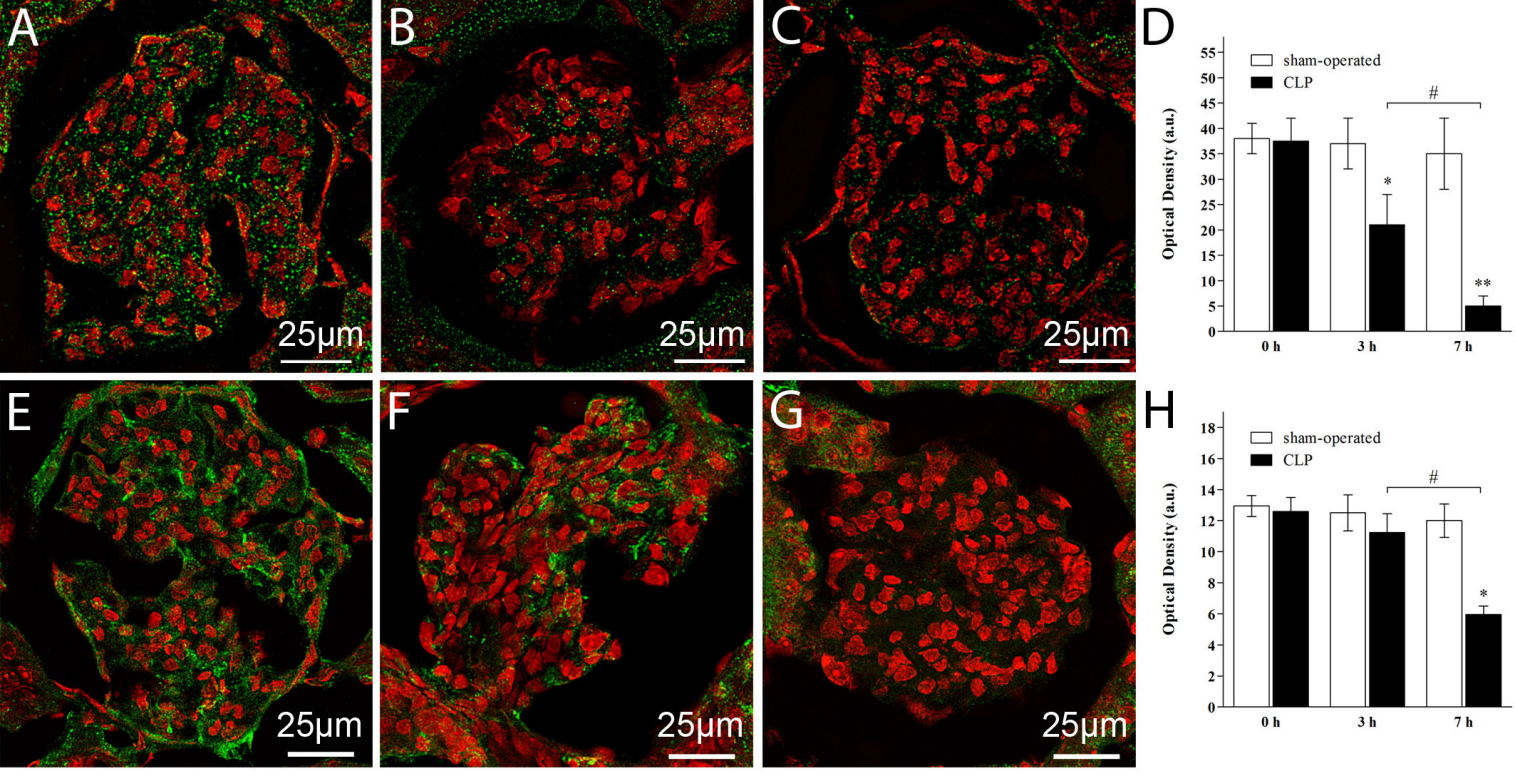
**Sepsis was associated with a reduction in the expression of Syndecan-1 and HA content in renal corpuscles**. **(A-D) **Confocal immunofluorescence of cryostat sections incubated with polyclonal antibody against Syndecan-1 (in green) and counterstained with propidium iodide (PI, red) to detect nuclei. Note the expression of Syndecan-1 (in green) in the renal corpuscle of sham-operated rats (panel A) and its progressive decrease over time in septic corpuscles (panel B at 3 hours and panel C at 7 hours; ** *P *<0.01, CLP versus sham-operated, * *P *<0.05, CLP versus sham-operated, # *P *<0.05 CLP at 7 hours versus CLP at 3 hours, panel D). (**E-H) **Confocal immunofluorescence of cryostat sections incubated with biotinylated HA binding protein (bHABP) and visualized with Alexa Fluor 488-conjugated streptavidin and PI. HA content (in green) was significantly reduced in the renal corpuscles of rats subjected to CLP at 7 hours. Differences between sham-operated and 3 hour-treated rats are negligible (panel E for sham-operated, panel F and G for CLP at 3 and 7 hours, respectively, * *P *<0.05, CLP versus sham-operated, # *P *<0.05 CLP 7 hours versus CLP 3 hours, panel H). CLP, cecal ligation and puncture.

Contrarily to the limited structural and ultrastructural changes found in the septic rats, alteration in glycocalyx-associated proteoglycans was diffuse and consisted in a reduction of syndecan-1 expression by 50% and 80%, respectively at 3 and 7 hours in the renal corpuscles of CLP-treated rats as compared to sham-operated (Figure 3, panel A for sham-operated, panel B and C for CLP at 3 and 7 hours respectively, panel D for quantitative analysis). Immunohistochemistry of HA based on bHABP decoration showed a significant reduction in the HA content in the corpuscles of CLP-treated rats at 7 hours only (Figure 3, panel E for sham-operated, panel F and G for CLP at 3 and 7 hours, respectively, panel H for quantitative analysis).

### Lectin reactivity location and intensity

#### MAA and SNA

**Figure 4:**
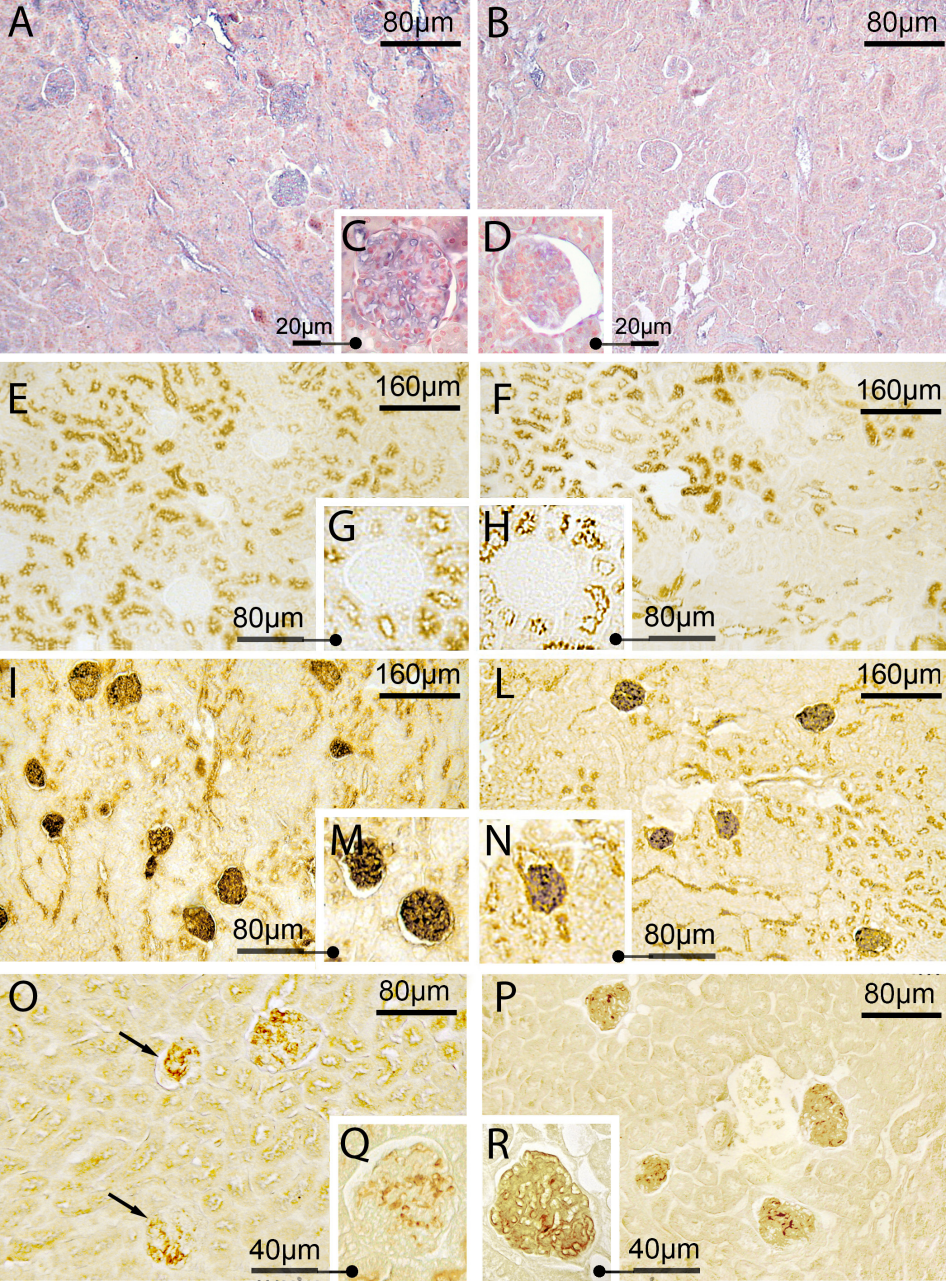
**Sepsis was associated with reduced expression and changes in chemical structure of glomerular sialic acids**. Representative light microphotograph of lectin histochemistry at 7 hours in sham-operated and CLP-treated rats. Panels **A-D**: Sialic acid linked α-2,6 to Gal/NAcGal was decreased during sepsis. SNA reactivity (detected by blue staining) was decreased in renal corpuscles of CLP (panels B and D) with respect to sham-operated rats (panels A and C). Panels **E-N**: Sialic acid linked to D-Gal(β-1,3)-D-GalNAc was decreased during sepsis. No direct reactivity to PNA was observable in either sham-operated (panels E and G) or septic rats (panels F and H). After neuraminidase digestion, PNA reactivity appeared (in brown) but with less intensity in CLP (panels L and N) than in sham-operated rats (panels I and M). Panels **O-R**: Sialic acids containing C_7 _and/or C_8 _and/or C_9_-O-acetyl groups in the side chain were increased after sepsis. After mild oxidation-neuraminidase treatments, PNA reactivity (in brown/yellow) appeared more intense in CLP than in sham-operated renal corpuscles. CLP, cecal ligation and puncture; PNA, peanut agglutinin (*Arachis hypogaea*); SNA, *Sambucus nigra *agglutinin.

**Figure 5:**
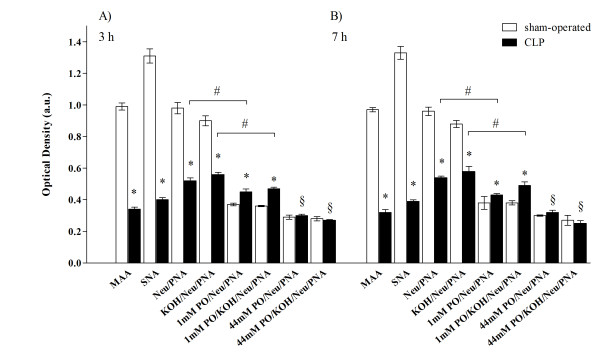
**Sepsis was associated with reduced expression and changes in chemical structure of GFB sialic acids**. Quantitative analysis of lectin histochemistry at 3 and 7 hours after sham or CLP operation. Reactivity intensity of MAA, SNA and PNA, after treatment with neuraminidase (with and without deacetylation), was significantly lower in CLP compared to sham-operated samples, demonstrating a decrease of sialic acid in septic rats. After mild oxidation-neuraminidase treatment (with and without deacetylation), PNA reactivity intensity was lower in sham-operated than in CLP rats, demonstrating a major amount of sialic acid with acetylic groups in C_7_- and/or C_8_- and/or C_9_- in septic rats. Data from mild oxidation were lower compared to those without mild oxidation for both sham-operated and CLP rats, indicating that some but not all sialic acids were acetylated in C_7_-and/or C_8_- and/or C_9_-. No significant difference between groups was observable in PNA reactivity intensity, after strong oxidation-neuraminidase treatment, with and without deacetylation; for both groups, reactivity intensity was statistically lower when compared to the neuraminidase PNA reactivity with and without mild oxidation, thus indicating that some sialic acids were acetylated in C_9 _and linked α-2,3. No difference between 3 and 7 hours was found. (* *P *<0.05 sham-operated versus CLP same reaction; # *P *<0.05 mild oxidation versus without oxidation; § *P *<0.05 strong versus mild and versus without oxidation, panels **A **and **B**). CLP, cecal ligation and puncture; MAA, Maackia amurensis agglutinin; PNA, peanut agglutinin (Arachis hypogaea); SNA, Sambucus nigra agglutinin; Neu/PNA, neuraminidase/PNA; KOH/Neu/PNA, deacetylation/neuraminidase/PNA; 1 mM PO/Neu/PNA, mild oxidation/neuraminidase/PNA; 1 mM PO/KOH/Neu/PNA, mild oxidation/deacetylation/neuraminidase/PNA; 44 mM PO/Neu/PNA, strong oxidation/neuraminidase/PNA; 44 mM PO/KOH/Neu/PNA, strong oxidation/deacetylation/neuraminidase/PNA.

MAA and SNA reactivity (which show the presence of sialic acids linked α-2,3 and α-2,6 to galactose/galactosamine with a direct method) was present in the GFB of both the sham-operated and CLP groups (for SNA see Figure 4, panels A, B, C and D). Quantitative analysis showed however that both reactivities were significantly lower in samples taken from CLP rats than those from sham-operated rats (Figure 5 panel A). No difference in the reduction of MAA and SNA reactivity between 3 and 7 hours was detected in septic rats (Figure 5 panel B).

#### PNA and PNA with enzymatic and chemical treatments

PNA reactivity was absent in the renal corpuscles taken from the sham-operated and septic rats (Figure 4, panels E, F, G and H for 7 hours after sham or CLP operation). After neuraminidase digestion (indirect method to show presence of sialic acid) with and without deacetylation (to show the presence of acetylic group on C_4 _of the pyranose ring of sialic acid), PNA reactivity appeared in the GFB of both study groups (Figure 4, panels I, L, M, and N for neuraminidase without deacetylation), although in the CLP group it was reduced compared to sham-operated (Figure 5, panel A). No significant difference in reactivity intensity was observed with and without deacetylation within each group, demonstrating the absence of acetyl groups on C_4 _(Figure 5).

After mild oxidation-neuraminidase treatment with or without deacetylation (which reveals C_7_- and/or C_8_- and/or C_9_-O-acetyl groups in the side chain of sialic acid), PNA reactivity was present in the GFB of both groups (Figure 4, panels O, P, Q and R) but the intensity was lower in sham-operated than in CLP rats, demonstrating a major amount of sialic acid acetylated in C_7_- and/or C_8_- and/or C_9 _in the GFB of septic rats (Figure 5 for quantitative analysis). When data from mild oxidation are compared to those without oxidation, reaction intensity was lower with than without mild oxidation both for sham-operated and CLP, indicating that some but not all sialic acids were acetylated in C_7_-and/or C_8_- and/or C_9 _(Figure 5).

After strong oxidation-neuraminidase treatment, with and without deacetylation (which reveals acetyl groups in C_9 _of sialic acid linked α-2,3 to galactose), PNA reactivity was present in the same amount in the GFB of both groups. For both groups, the intensity of this reactivity was statistically lower when compared to the neuraminidase PNA reactivity with and without mild oxidation (Figure 5), thus indicating that some sialic acids were acetylated in C_9 _and linked α-2,3.

There were no differences in these reactivities between 3 and 7 hours (Figure 5).

Altogether, our results indicate a massive decrease in total amount of sialic acid and a parallel increase of the acetylation of the remaining sialic acid residues in the GFB. These alterations are present in all the examined renal corpuscles of septic rats.

#### Controls

Sections incubated with lectins and their corresponding hapten sugars and sections incubated with unconjugated lectins, were unstained. Sections incubated with enzyme-free buffer did not show any change in lectin binding. Results of the efficacy of enzymatic digestion were as expected. The desulfation procedure did not prove to affect the subsequent lectin-binding.

## Discussion

Our results demonstrate that during the initial phases of sepsis renal damage occurs and it consists of structural and ultrastructural alterations of some renal corpuscles and diffuse alteration of the glycocalyx components of the GFB which leads to increased permeability to albumin.

To induce sepsis, we chose the CLP model, a systemic polymicrobial infection of intestinal origin, which is considered the 'gold standard' for experimental sepsis [42] in that it mimics the cardinal clinical features, the hemodynamic and metabolic phases and the development of multi-organ dysfunctions typical of human sepsis [46]. The CLP-treated animals showed the typical clinical signs such as the proliferation of microorganisms in the peritoneal fluid and TNF-α was increased in blood collected from remote vessels, indicating the systemic spread of the inflammatory response. No mortality was observed in our study, in accordance with the short time course of the experimental protocol whereas in the CLP model in our laboratory mortality (which ranges between 15% and 20%) usually starts after 12 hours and peaks between 24 and 48 hours [47].

The CLP model has been associated with the development of acute kidney injury (AKI) in some studies but not in all [48]. It should be pointed out that our aim was not to create a model of AKI, but rather to observe those changes in the kidney, and specifically to vascular permeability in GFB, which are likely to occur during the initial phases of sepsis, when the effects of increased vascular permeability are major and biochemical markers indicating kidney damage are not yet increased. We observed alteration in the main components of GFB associated glycocalyx, that is the sialic acids, syndecan-1 and HA - associated with loss of GFB perm-selectivity (documented by albumin leakage into urine). Interestingly, the sialic acid content of GFB was not only reduced but also changed, as it showed a higher degree of acetylation, specifically the amount of sialic acids with acetyl groups C_7_-and/or C_8 _linked α-2,3 and α-2,6 to galactose, and/or C_9 _linked α-2,6 to galactose in the side chain. O-acetylation, in particular in C_9 _position, has a specific role in defense against bacterial neuraminidase [20] and can be involved in the 'masking of recognition sites' exerted by sialic acids [21]. Therefore, if we imagine that endothelial cells and podocytes of the GFB are not only spectators but also actors of the response to sepsis, we might speculate that the increase in acetylic groups is a compensatory mechanism attempting to prevent further desialylation of glycocalyx and limiting the action of circulating pro-inflammatory molecules during sepsis. Thus, acetylation of sialic acids - as an endogenous response or pharmacological intervention - might be viewed as a strategy to preserve the GFB and its functionality during infections.

Our findings, that link GFB glycocalyx disruption and albuminuria, are in agreement with data from experimental studies using models of genetic mutants, induced nephrosis, diabetes and glomerular injury after desialylation [26-28,30,49-51] in which sialic acids have been documented to act as key regulators of the GFB architecture and functionality. GFB glycocalyx components rich in anions, especially sialic acids, prevent the passage of anionic protein such as albumin into urine under physiological conditions and thus are part of the so-called 'charge barrier [7,30].

In view of our findings showing increased TNF-α plasma levels and the occurrence of a severe intra-peritoneal infection in the septic rats, it is likely that the mechanisms underlying glycocalyx disruption involve TNF-α increase and endotoxin release, in agreement with previous studies in the literature [52].

Few studies have examined the relationship between glycocalyx dysfunction and sepsis in humans. While no study specifically addressing the relationship between vascular HA turnover and sepsis is available, there are data showing that GAGs and syndecan-1 circulating levels increase in septic shock patients, reflecting the shedding of glycocalyx proteoglycans, and they are correlated with mortality and organ dysfunction, respectively [53]. Dosing in plasma or in urine molecules indicating glycocalyx turnover has been suggested to be a marker of sepsis [54,55]. However, in the human studies reported above, the authors could only speculate that GAGs and syndecan-1 came from the endothelial glycocalyx, because they did not provide direct evidence (morphological or structural) of the origin of these molecules. This lack of evidence is a major limitation, since plasma GAGs may have multiple origins, such as from broken or damaged tissues with high connective content. By contrast, one of the major points of our study is that we provided strong - structural, biochemical and histochemical - evidence of GFB glycocalyx damage associated with functional impairment of the GFB in sepsis.

The relationship between glycocalyx and permeability is under study. Destruction of the glycocalyx, using enzymatic approaches, leads to increased capillary permeability [56]. Also inflammation, such as that occurring after ischemia-reperfusion, causes disruption of glycocalyx and an increase in permeability [8,56]. Albuminuria is specifically due to GFB damage, as it is considered 'selective glomerular proteinuria' in contrast to the low molecular weight proteinuria that is generally due to tubular abnormalities [57]. In our experimental conditions, the presence of albumina in the urine strengthens the idea that alterations of the GFB function may represent an initial event of sepsis, even though damage to the tubular components (which fail to reabsorb proteins with lower molecular weight than albumin) cannot be excluded.

This study has some limitations, such as the duration of the experimental time course. However, whereas in human patients the onset and progression of sepsis occurs over days to weeks, in the CLP model the development of sepsis occurs in hours to days. Therefore, we maintain that the experimental time chosen in the present study, although relatively short (up to 7 hours only), is sufficient to reproduce the initial phases of sepsis in a clinically relevant way [42,46,58]. It would also be interesting to observe what happens to the GFB after a longer time and identify therapeutic strategies and interventions aimed at favoring the healing of GFB associated glycocalyx. This latter might also provide a causative link between GFB glycocalyx morphological damage and GFB dysfunction (albuminuria) which at present has not been determined. Another limitation of our study is that we have focused our attention on the GFB alterations only. However, we do not exclude the possibility that other components, such as the tubular structures of the renal cortex and other vascular components, may also be seriously damaged as well. Experiments are ongoing in our laboratory to clarify these points.

Despite these limitations, we provide evidence that sepsis, in its early development, is associated with loss of the perm-selectivity of the GFB, as documented by leakage of albumin into urine. At the molecular level, perturbation of GFB glycocalyx also occurs, with a decrease and change in the amount and conformation of sialic acids and a decrease in syndecan-1 and HA. Whether restoration of glycocalyx components might represent a new, very appealing therapeutic approach to kidney injury during sepsis, remains to be elucidated.

## Conclusions

In its initial phase, sepsis is associated with a significant alteration in the composition of the GFB-associated glycocalyx, with loss of GFB perm-selectivity, as documented by albumin leakage into urine. Restoration of glycocalyx components might represent a potential therapeutic approach to maintain GFB function during sepsis.

## Key messages

• The regulation of vascular permeability is a complex phenomenon which has not been completely clarified yet. Recently, attention has been devoted to the glycocalyx as one of the main element contributing to it.

• A relationship between glycocalyx dysfunction and increased vascular permeability during sepsis has been suggested by some studies, but none has specifically addressed this issue in the glomerular filtration barrier (GFB).

• In a clinically relevant, controlled rat model of polymicrobial sepsis (the Cecal Ligation and Puncture model) changes in structural, ultrastructural and biochemical composition of the GFB, together with loss of its GFB perm-selectivity were investigated.

• Sepsis is associated in its initial phase with a significant alteration in the composition of the GFB-associated glycocalyx, with loss of GFB perm-selectivity as documented by albumin leakage into urine.

• Restoration of glycocalyx components might represent a new therapeutic approach to maintain GFB function during sepsis.

## List of abbreviations

AKI: acute kidney injury; bHABP: biotinylated HA binding protein; BSA: bovine serum albumin; CLP: cecal ligation and puncture; DIG: digoxigenin; GAG: glycosaminoglycan; GFB: glomerular filtration barrier; HA: hyaluronan; HRP: horseradish peroxidase; MAA: *Maackia amurensis *agglutinin; n: number; Neu/PNA: neuraminidase/PNA; NIH: National Institutes of Health; OD: optical density; PBS: phosphate-buffered saline; PNA: peanut agglutinin (*Arachis hypogaea*); ROI: region of interest; SDS: sodium dodecyl sulfate; SEM: standard error of the mean; SNA: *Sambucus nigra *agglutinin; TEM: transmission electron microscopy.

## Competing interests

The authors declare that they have no competing interests.

## Authors' contributions

CA designed, coordinated and supervised all experiments, analyzed the data and drafted the manuscript. ES supervised the histological procedures and drafted and revised the manuscript. LV and VS carried out the animal experiments, were involved in the data collection and helped in writing the manuscript. MM, AT and LB performed optical, confocal and electron microscopy. DN was responsible for image analysis. ALC performed biochemical essays {AU Query: Would assays or tests be a better word here than essays? http://tests is a better word}. LF participated in the study design and drafted and revised the manuscript. ARDG conceived the study and participated in its design. All authors read and approved the final manuscript.

## Supplementary Material

Additional file 1 **pdf - "Lectin Histochemistry"**. A pdf file containing detailed information on lectin histochemistry. Click here for file
