# Supplementary material for: Sepsis induces albuminuria and alterations in the glomerular filtration barrier: a morphofunctional study in the rat
Source: Crit Care. 2011 Nov 22;15(6):R277. doi: 10.1186/cc10559 (PMC3388657; doi:10.1186/cc10559)
Supplement: Additional file 1 — pdf - "Lectin Histochemistry". A pdf file containing detailed information on lectin histochemistry. [file cc10559-S1.PDF]

Lectin Histochemistry

Two methodologies were used for lectin histochemistry: the "direct" technique and the "indirect" one. The first one uses MAA and SNA to identify and differentiate between sialic acids linked α-2,3 and α-2,6 to galactose or galactosamine respectively (Neu5Ac(α-2→3)Gal, Neu5Ac(α-2→6)Gal/GalNAc) [1-4]. MAA and SNA were digoxigenin (DIG) labeled lectins and purchased from Roche Diagnostic GmbH (Mannheim, Germany). In the second methodology PNA, combined with neuraminidase digestion, deacetylation and differential oxidation to reveal acetylic groups, is used to investigate the expression of sialic acid linked to D-Gal(β1→3)-D-GalNAc, and the structure of sialic acids [3-4]. PNA was horseradish peroxidise (HRP) conjugated lectin, obtained from Sigma-Aldrich (St. Louis, MO, USA). HRP-conjugated and DIG-labeled lectin use were carried out as reported in previous studies [4-5].

In some experiments, sialic acid linked to α-2,3, α-2,6 and α-2,8 was removed by pretreating the sections with neuraminidase (Type X from *Clostridium perfringens*; specific for sialic acids linked α-2,3, α-2,6 and α-2,8; Sigma-Aldrich, St. Louis, MO, USA), before staining with HRP-conjugated PNA. Deacetylation was performed by incubation of the sections with KOH in ethanol. This treatment renders sialic acid residues, which also contain acetylic groups on C_4 of the pyranose ring, susceptible to neuraminidase digestion. 1 mM aqueous periodic acid (1 mM PO, mild oxidation) and 44 mM aqueous periodic acid (44 mM PO, strong oxidation) were used for differential oxidation. Mild oxidation abolishes the staining with neuraminidase/PNA or KOH/neuraminidase/PNA when sialic acid does not contain C_7-and/or C_8- and/or C_9-O-acetyl groups in the side chain. Strong oxidation blocks the subsequent staining with neuraminidase/PNA or KOH/neuraminidase/PNA except for C_9 acetylated sialic acids linked α-2,3 bound to the penultimate β-galactose [3-4].

Controls for lectin specificity included substitution of lectin-conjugates with the respective unconjugated lectins or preincubation of lectins with the corresponding hapten sugars: 0.1 M α-2,3 sialyllactose for MAA, 0.1 M α-2,6 sialyllactose for SNA, 0.2 M D-galactose for PNA (Sigma-

Aldrich, St.Louis, MO, USA). Control of sialidase digestion was made by incubation of the sections with enzyme-free buffer. The efficacy of digestion was tested by treating adjacent sections, with and without prior deacetylation, with the enzyme solution and then submitting them to MAA and SNA labeling. Some control sections were treated with a desulfation procedure. This procedure eliminates sulfated groups present on the carbohydrate chains that could interfere with lectin binding [3-4].

Evaluation of reactivity location and its intensity

In each section (3 sections per specimen), the staining location was examined and densitometric analysis of histochemical data was performed by measuring the average optical density (OD) on regions of interest (ROI, 40 μm^2 area) of light and confocal microscopy images, using ImageJ software. Measured values were normalized to background [(OD-ODbkg)/ODbkg]. At least 8 regions of interest in 10 different optical fields were analyzed in each experiment and the mean ± SEM OD was then calculated.

References

1. Shibuya N, Goldstein IJ, Broekaert WF, Nsimba-Lubaki M, Peeters B, Peumans WJ: **Fractionation of sialylated oligosaccharides, glycopeptides, and glycoproteins on immobilized elderberry (Sambucus nigra L.) bark lectin.** *Arch Biochem Biophys* 1987, **254:**1-8.
2. Wang WC, Clark GF, Smith DF, Cummings RD: **Separation of oligosaccharides containing terminal alpha-linked galactose residues by affinity chromatography on Griffonia simplicifolia I bound to concanavalin A-sepharose.** *Anal Biochem* 1988, **175:**390-396.
3. Gabrielli MG, Bondi AM, Materazzi G, Menghi G: **Differential location and structural specificities of sialic acid-beta-D-Gal sequences belonging to sialoderivatives of rabbit oviduct under hormonal treatment.** *Histol Histopathol* 2004, **19:**1175-1186.
4. Mencucci R, Marini M, Gheri G, Vichi D, Sarchielli E, Bonaccini L, Ambrosini S, Zappoli Thyrion GD, Paladini I, Vannelli GB, Sgambati E: **Lectin binding in normal, keratoconus and cross-linked human corneas.** *Acta Histochem* 2010.
5. Sgambati E, Marini M, Vichi D, Zappoli Thyrion GD, Parretti E, Mello G, Gheri G: **Distribution of the glycoconjugate oligosaccharides in the human placenta from pregnancies complicated by altered glycemia: lectin histochemistry.** *Histochem Cell Biol* 2007, **128:**263-273.
